# Establishment of human Leber’s hereditary optic neuropathy model using iPSC-derived retinal organoids

**DOI:** 10.3389/fncel.2025.1635775

**Published:** 2025-09-12

**Authors:** Kota Aoshima, Yuya Takagi, Michinori Funato, Yoshiki Kuse, Shinsuke Nakamura, Masamitsu Shimazawa

**Affiliations:** 1 Molecular Pharmacology, Department of Biofunctional Evaluation, Gifu Pharmaceutical University, Gifu, Japan; 2 Department of Clinical Research, NHO, Nagara Medical Center, Gifu, Japan

**Keywords:** retinal organoid, Leber’s hereditary optic neuropathy, mitochondrial disease, in vitro disease modeling, mitophagy

## Abstract

Leber’s hereditary optic neuropathy (LHON) is a mitochondrial disease caused by mitochondrial DNA mutations, leading to central vision loss and retinal ganglion cell (RGC) degeneration. Progress in understanding LHON and developing treatments has been limited by the lack of human-like models. In this study, we aimed to establish a human retinal model of LHON using retinal organoids (ROs) from LHON patient-derived induced pluripotent stem cells (LHON-iPSCs). We first confirmed LHON-iPSCs were successfully differentiated into ROs (LHON-ROs). LHON-RO showed a reduction in RGC numbers and the density of neural axons. Additionally, both mitochondrial membrane potential and ATP production were decreased in LHON-RO. Finally, treatment with idebenone, the only approved therapeutic agent for LHON, improved RGC numbers in LHON-RO. This model replicates key clinical features of LHON, including RGC and axonal loss, and demonstrates idebenone’s therapeutic potential. Furthermore, a comprehensive analysis of the LHON-RO model revealed impaired mitophagy, suggesting novel therapeutic targets for LHON. Thus, the LHON-RO model offers a valuable platform for studying LHON pathogenesis and evaluating treatments.

## 1 Introduction

Mitochondrial diseases are disorders caused by mutations in mitochondrial DNA (mtDNA). Leber’s hereditary optic neuropathy (LHON) is a mitochondrial disease characterized by blindness due to the death of retinal ganglion cells (RGCs), which form the optic nerve (Levin, 2007). More than 90% of LHON patients have one of three common mtDNA mutations: 11778G>A, 14484T>C, or 3460G>A (Yen et al., 2006; Tun et al., 2014). These mutations occur in the coding regions for ND4, ND6, and ND1, respectively, which are components of mitochondrial complex I. The mutations reduce the activity of the mitochondrial respiratory chain complex I subunit (Hofhaus et al., 1996; Pello et al., 2008; Jiang et al., 2016), leading to the accumulation of reactive oxygen species (ROS) and decreased adenosine triphosphate (ATP) production, resulting in RGC degeneration (Danielson et al., 2002; Zanna et al., 2005).

Currently, there are two major challenges in the treatment of LHON. First, the pathogenesis of LHON is not yet fully understood, especially the mechanism of RGC-specific damage due to mitochondrial mutations. Second, therapeutic options are limited, with idebenone, a coenzyme Q10 analog (Yu-Wai-Man et al., 2024), being the only approved treatment. However, idebenone has been reported not to improve vision in some patients (Culea et al., 2019), and it is approved only by the Committee for Medicinal Products for Human Use of the European Union. As a result, a limited number of patients have access to this drug, and some patients do not respond to it. Therefore, there is an urgent need to better understand the pathogenesis of LHON and identify new therapeutic agents.

Until now, some researchers conduct to develop a new therapeutic agent for LHON (Hage and Vignal-Clermont, 2021). However, the development of new therapeutic agents have not yet progressed due to inadequate evaluation systems, which means there are no models of the disease, including genetically modified animals, because LHON is caused by mtDNA mutations. Although there is a model of retinal damage induced by rotenone, an inhibitor of the mitochondrial respiratory chain complex I subunit, it has not been able to correctly mimic LHON pathology (Heitz et al., 2012; Mansergh et al., 2014; Aoyama et al., 2021). Other studies have focused on LHON patient-derived cells in culture, including fibroblasts (FBs) and induced pluripotent stem cells (iPSC)-RGCs, where patient iPSCs are differentiated into RGCs (Wu et al., 2018; Yang et al., 2019, 2020; Zhou et al., 2022; Aleo et al., 2024). These models are valuable for examining mitochondrial abnormalities in the pathogenesis of LHON. Some researchers have successfully produced LHON-retinal organoids (ROs) (Ueda et al., 2021; Kurzawa-Akanbi et al., 2024). However, these models have not yet mimicked the clinical findings in humans with LHON, such as the reduced density of RGCs and nerve axons associated with thinning of the retinal neurofilament layer (RNFL) of the retina. Therefore, to elucidate the pathogenesis of the disease, the establishment of a stable RO model evaluation system is urgently needed. In this study, we aimed to construct the LHON-RO model and identify the novel therapeutic targets on LHON.

## 2 Methods

### 2.1 Ethical considerations

The research followed the principles outlined in the Declaration of Helsinki. The procedures for pathological analyses and the establishment of patient-derived iPSCs, including human gene analyses, were approved by the Ethics Review Committee of the National Hospital Organization, Nagara Medical Center (Approval Nos. 2018-20, 2019-18, 2019-23, 2019-38, 2020-20, 2021-7, 2021-22, 2024-24) and Gifu Pharmaceutical University (Approval Nos. 30-39, 2-14, 2-29, 3-21, 3-35), Japan. The established human iPSCs were handled in accordance with the revised guidelines for clinical research using human stem cells issued by the Ministry of Health, Labor, and Welfare of Japan.

### 2.2 iPSC culture and differentiation of ROs

The iPSCs were cultured following previously described protocols (Ohuchi et al., 2019). We used iPSCs derived from a control individual (wild-type [WT]-iPSCs, 201B6 line) and from a patient with LHON (mtDNA 11778G>A) (LHON-iPSCs, #1: HPS1900, #2: HPS1776). iPSC line (201B6) derived from skin fibroblasts, established at and provided from the Center for iPS Cell Research and Application, Kyoto University.(Takahashi et al., 2007) iPSC lines (HPS1900, HPS1776) derived from peripheral blood mononuclear cells, were purchased from the RIKEN Cell Bank (Table 1). Informed consent was obtained from all subjects or their legal guardians. These iPSCs are not infected, and are STR-matched to the patient. iPSC colonies were maintained in 5% CO_2_ at 37 °C and passaged every 7 days. iPSCs at passages 30 to 45 were used. A modified protocol for generating normal ROs was used (Capowski et al., 2019). iPSCs were differentiated into ROs using the following procedure: a serum-free floating culture of embryoid body (EB)-like aggregates and the quick reaggregation (SFEBq) method. On Day −1, 10,000 single iPSCs were seeded with 10 μM Rho-associated coiled-coil forming kinase inhibitor Y-27632 (Wako, Osaka, Japan) in embryonic stem cell medium (ESCM). The iPSCs were seeded onto pluronic F127-coated 96 U bottom well plates. On Day 0, differentiation into ROs was initiated. The culture medium was replaced with 2 μM dorsomorphin (Sigma-Aldrich Corp., St. Louis, MO, USA) and 10 μM SB431542 (Cayman Chemical, Ann Arbor, MI, USA) in embryoid body medium (EBM), which consists of Dulbecco’s modified Eagle’s medium + GlutaMAX (DMEM)/F12 (Life Technologies, Carlsbad, CA, USA), 20% knockout serum replacement (Life Technologies), 1% nonessential amino acids (NEAA), 0.1% 2-mercaptoethanol, and 500 U/mL Penicillin–Streptomycin (PS). On Day 3, neural induction medium (NIM), consisting of DMEM/F12 + GlutaMAX, 1% NEAA, 1% N2 supplement (Life Technologies), and 500 U/mL PS, was added to the EBM. On Day 7, the EBs were transferred to Matrigel-coated plates (30 EBs per well on a 6-well plate) (Becton, Dickinson and Company, Franklin Lakes, NJ, USA), and the culture medium was replaced with fresh NIM containing 50 ng/mL bone morphogenetic protein-4 (BMP-4) (R&D Systems, McKinley Place, MN, USA). On Day 10, half of the medium was replaced with fresh NIM. Then, on Day 13, the entire medium was replaced with fresh NIM, gradually lowering the BMP-4 concentration. On Day 14, the medium was replaced with retinal differentiation medium (RDM), which consisted of DMEM/F12 + GlutaMAX, 1% NEAA, 2% B27 supplement (Life Technologies), and 500 U/mL PS. Three-dimensional optic vesicles were observed approximately 20–30 days after EB aggregation. On Day 27, these optic vesicles were mechanically lifted using a Nanopass 34-gauge needle (Terumo Corporation, Tokyo, Japan). The organoids were cultured on pluronic F127-coated 96 U bottom well plates (one organoid per well), with media replaced every other day using 3D-RDM. This medium consisted of RDM supplemented with 5% fetal bovine serum, 100 μM taurine (Tokyo Chemical Industry Co., Ltd.), and 1 μM all-trans retinoic acid (Sigma-Aldrich Corp.).

**Table 1:** The information of iPSCs.

Information	LHON #1	LHON #2
Resorce No.	HPS1900	HPS1776
Age	60s	20s
Sex	Male	Male
Race	Japanese	Japanese
mtDNA mutation	11778G > A	11778G > A
Symptomatic	symptomatic	symptomatic

### 2.3 Immunocytochemistry

To prepare frozen sections, ROs were fixed with 4% paraformaldehyde (PFA) (Wako) and then stepwise replaced with 20 and 30% sucrose solutions. Subsequently, the ROs were embedded in optimal cutting temperature compound, and sections were cut at a thickness of 11 μm. Immunocytochemical staining was performed by blocking the samples in 10% horse serum in PBS containing 0.1% Triton-X for 1 h at room temperature. Primary antibodies (Table 2) were diluted in 10% horse serum and applied overnight at 4 °C. The following day, samples were washed in PBS, and secondary antibodies (Table 2) and Hoechst 33342 were diluted 1:1000 in 10% horse serum and applied for 1.5 h at room temperature. Finally, the samples were washed with PBS and mounted onto slides for imaging. Images were captured using a confocal fluorescence microscope (FLUO-VIEW FV10i; Olympus, Tokyo, Japan). RGC counts, measurements of neuroaxonal density, TUJ1 fluorescence intensity, Brn3 positive area and Chx10 positive area were performed in a blinded manner using ImageJ analysis software (National Institutes of Health).

**Table 2:** Primary antibodies and secondary antibodies used (immunocytochemistry).

Primary antibody	Company	Catalog number	Dilution
Brn3	Santa Cruz	sc-6026	1:50
Chx10	Santa Cruz	sc-365519	1:100
TUJ1	GeneTex	GTX130245	1:200

Secondary antibody	Company	Catalog number	Dilution
Donkey anti goat Alexa fluor 647	Invitrogen	A21447	1:1,000
Donkey anti rabbit Alexa fluor 546	Invitrogen	A10040	1:1,000
Donkey anti mouse Alexa fluor 488	Invitrogen	A21202	1:1,000

### 2.4 Whole-mount immunostaining and clearing

ROs were fixed in 4% PFA (Wako) at 4 °C overnight and then washed with 0.1% Tween in PBS. To increase antibody permeability into the tissues, the detergent CHAPS (3-[(3-cholamidopropyl)dimethylammonio]-1-propanesulfonate)/NMDEA (N-methyldiethanolamine) was used (Zhao et al., 2020). The samples were incubated for 1 h at 37 °C, washed with 0.1% Tween in PBS, blocked with Protein Block Serum Free (Dako), and incubated with primary antibodies (Table 2) diluted in Protein Block in PBS (1:10 dilution) for 7 days at 4 °C. After washing, fluorescent dye-conjugated secondary antibodies (Table 2) were applied to the samples overnight at 4 °C.

For clearing, the samples were dehydrated in 50% (vol/vol) methanol/PBS and then transferred to 100% methanol. The methanol was gradually replaced with 50% (vol/vol) benzyl alcohol/benzyl benzoate (BABB)/methanol and finally with 100% BABB (Yokomizo et al., 2012).

### 2.5 RO size measurement

Images were captured using a microscope (ZEISS Primovert). RO size measurements were performed automatically using ImageJ analysis software (National Institutes of Health) and OrgM (A Fiji macro for automated measurement of object area, diameter and roundness from bright field images).

### 2.6 RT-PCR analysis

ROs were incubated in a humidified atmosphere of 5% CO_2_ at 37 °C. After incubation, RNA was extracted using NucleoSpin RNA ІІ (Takara Bio Inc., Kusatsu, Japan). Real-time RT–PCR was performed using a Thermal Cycler Dice Real-Time System III (TP-950; Takara Bio Inc.) with SYBR Premix Ex Taq II (Takara Bio Inc.), according to the manufacturer’s protocol. The PCR primer sequences used are shown in Table 3. The results are expressed relative to the GAPDH internal control.

**Table 3:** Primer used in the study.

BRN3A Forward	AGTACCCGTCGCTGCACTCCA
BRN3A Reverse	TTGCCCTGGGACACGGCGATG
TUBB Forward	CTGGACCGCATCTCTGTGTACT
TUBB Reverse	GCCAAAAGGACCTGAGCGAACA
MT-ND4 Forward	ACAAGCTCCATCTGCCTACGACAA
MT-ND4 Reverse	TTATGAGAATGACTGCGCCGGTGA
MT-COX1 Forward	TCTCAGGCTACACCCTAGACCA
MT-COX1 Reverse	ATCGGGGTAGTCCGAGTAACGT
MT-ATP6 Forward	CCAATAGCCCTGGCCGTAC
MT-ATP6 Reverse	GCTTCCAATTAGGTGCATGA
GAPDH Forward	CCTGCACCACCAACTGCTTA
GAPDH Reverse	GGCCATCCACAGTCTTCTGAG

### 2.7 TUNEL assay

TUNEL assay was performed on day 35 to detect cell death using Roche Diagnostics GmbH (Mannheim, Germany) *In Situ* Cell Detection Kit, TMR red after Brn3 immunostaining with control-RO and LHON-RO. The RO sections were preincubated with 0.1% critic acid in milliQ containing 0.1% Triton X-100 (Bio-Rad, CA, USA) for 10 min at room temperature. TUNEL mixture was treated to RO sections for 60 min at 37 °C. Hoechst 33342 was added to the sections for 15 min at room temperature. Finally, they were mounted in a solution (Fluoromount; Diagnostic BioSystems, Pleasanton, CA, USA). Images were captured using a confocal fluorescence microscope (FLUO-VIEW FV10i; Olympus, Tokyo, Japan). RGC counts, the number of TUNEL positive cells and the number of TUNEL positive RGCs were performed in a blinded manner using ImageJ analysis software (National Institutes of Health).

### 2.8 MT1 assay

ROs were cultured in MT1-Dye (1:500) (Dojindo, Kumamoto, Japan) in 3D-RDM for 2 days (5% CO_2_, 37 °C). Frozen sections were then prepared as described in Section 2.4 (Immunocytochemistry). The sections were thawed, nuclear-stained, and mounted. Images were captured using a confocal fluorescence microscope (FLUO-VIEW FV10i; Olympus, Tokyo, Japan). The fluorescence intensity of MT1 was measured in a blinded manner using ImageJ analysis software (National Institutes of Health).

### 2.9 ATP assay

ROs were incubated in a humidified atmosphere of 5% CO_2_ at 37 °C. On Day 35, ROs were transferred one by one to black μClear® plates. ATP levels were determined using the ATP Assay Kit-Luminescence (Dojindo, Kumamoto, Japan).

### 2.10 Gene expression analysis

Public RNA-seq datasets of FBs from LHON patients and non-LHON patients were analyzed using the NCBI Gene Expression Omnibus (accession number: GSE144914) (Lopez Sanchez et al., 2020). The gene sets used for mitochondrial respiratory chain complex I assembly (GO:0032981) and mitochondrial proton-transporting ATP synthase complex assembly (GO:0033615) were referenced from Mouse Genome Informatics. Gene set enrichment analysis (GSEA) was performed with the javaGSEA application (version 4.3.3)^1^ using the ‘weighted’ enrichment statistics setting (Mootha et al., 2003; Subramanian et al., 2005). To compare our data with the expression study by Lopez et al., data from the previous study were analyzed using gene sets related to mitochondrial complexes, such as mitochondrial complex (all) (GOCC MITOCHONDRIAL PROTEIN CONTAINING COMPLEX), mitochondrial complex I (HP DECREASED ACTIVITY OF MITOCHONDRIAL COMPLEX I), mitochondrial complex III (HP DECREASED ACTIVITY OF MITOCHONDRIAL COMPLEX III), and mitochondrial complex V (GOMF PROTON TRANSPORTING ATP SYNTHASE ACTIVITY ROTATIONAL MECHANISM). Enrichment was considered significant if the FDR was below 0.2 and the *p*-value was below 0.05.

### 2.11 Idebenone treatment

ROs were incubated in a humidified atmosphere of 5% CO_2_ at 37 °C. On Day 35, the medium was replaced with idebenone (TCI, Tokyo, Japan) -containing medium. The final concentrations of idebenone were 2 or 10 μM, with DMSO as the solvent (final concentration: 0.1%). The medium was changed every other day. Sampling was performed 7 days after idebenone treatment.

### 2.12 RNA sequencing (RNAseq)

Total RNA was extracted from Control-RO and LHON#1-RO at Day35 using NucleoSpin RNA ІІ (Takara Bio Inc., Kusatsu, Japan). The duality of the extracted RNA was assessed using an Agilent 2,100 Bioanalyzer (Agilent Technologies). RNA samples with RNA integrity number >8.0 were subjected to RNA-Seq analysis. RNA libraries were prepared from 50 ng of total RNA using the SMART-Seq v4 Ultra Low Input RNA Kit (Takara Bio, Mountain View, CA, USA), according to the manufacture’s instructions. The prepared libraries were sequenced on a NovaSeq 6,000 devise (Illumina, San Diego, CA, USA) using paired-end 150-bp reads. The sequencing reads were aligned to GRCm38 using a RefSeq annotation. Control analysis using Takara Bio provided the QC metrics and normalized read counts per gene. Differential expressed genes (DEGs) were extracted with an adjusted fold change > 1.5 using the Subio platform (Subio, Kasugai, Japan). Functional enrichment analysis was performed using the Database for Annotation, Visualization, and Integrated Discovery to identify the over-represented Gene Ontology (GO) terms among the differentially expressed genes. Gene set enrichment analysis (GSEA) was also performed using LHON-RO datasets.

### 2.13 Western blotting analysis

ROs were incubated in a humidified atmosphere of 5% CO_2_ at 37 °C. After incubation, recovery of cell lysates, measurement of protein concentrations, and confirmation of the immunoreactive bands were performed with reference to this previous study (Inagaki et al., 2025). The primary and secondary antibodies used are listed in Table 4.

**Table 4:** Primary antibodies and secondary antibodies used (western blotting analysis).

Primary antibody	Company	Catalog number	Dilution
PINK1	CST	#6946	1:1,000
Parkin	CST	#4211	1:1,000
LC3B	CST	#3868	1:1,000
p62	CST	#8025	1:1,000
β-actin	Millipore	A2228	1:1,000

Secondary antibody	Company	Catalog number	Dilution
Goat anti-Rabbit IgG (H + L) HRP	Thermo Fisher	#32460	1:500
Goat anti-Mouse IgG (H + L) HRP	Thermo Fisher	#32430	1:500

### 2.14 Quantification and statistical analysis

All key experiments were performed with at least two independent differentiation rounds and four technical repeats per condition and per line. Data are presented as means ± standard errors of the means (SEMs). Statistical comparisons were made using Student’s *t*-test (two-tailed) or Dunnett’s test with SPSS Statistics software (IBM, Armonk, NY, USA). The level of statistical significance was set at *p* < 0.05.

## 3 Results

### 3.1 Creation of ROs from LHON-iPSCs

**Figure 1:**
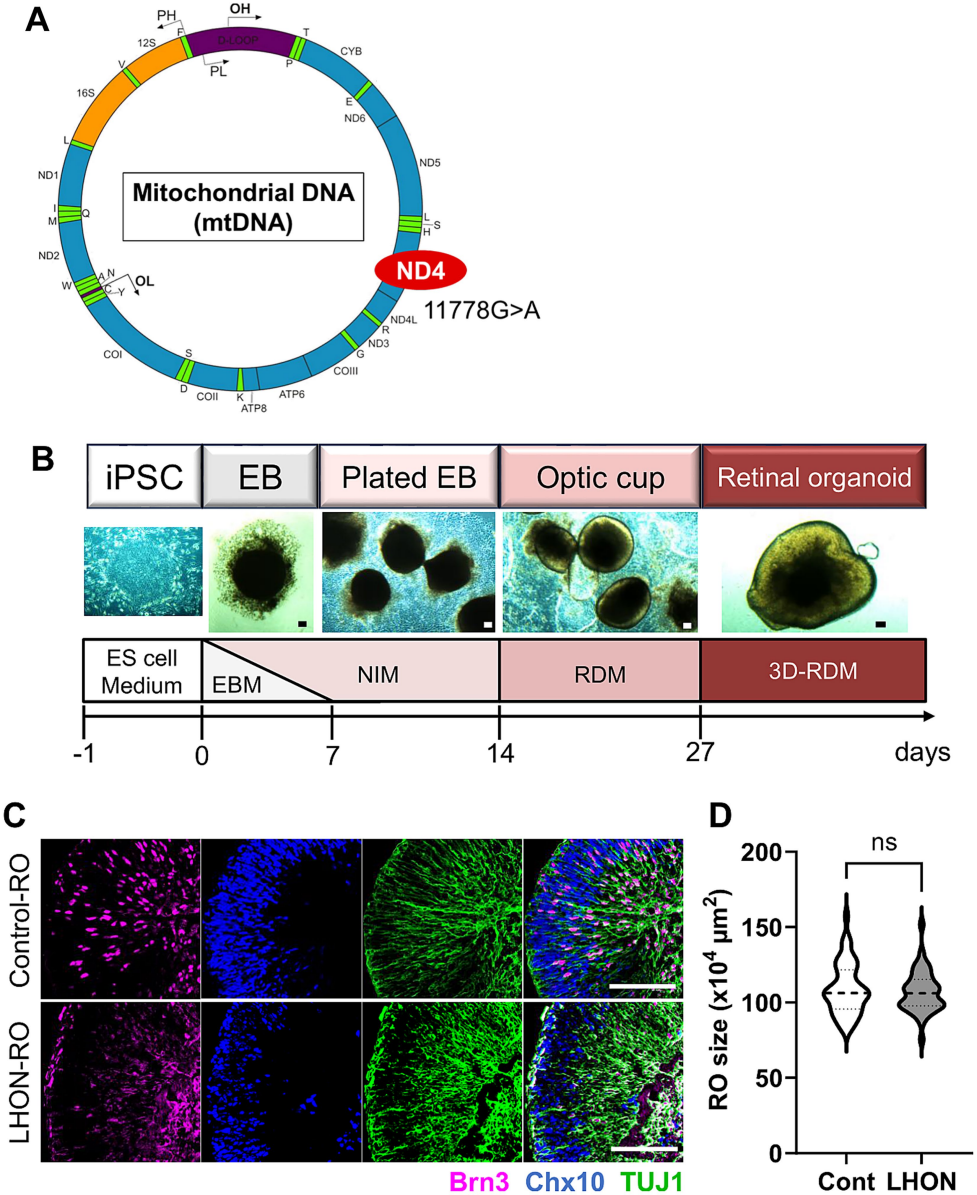
Protocol for RO differentiation. **(A)** Schematic of mitochondrial DNA and mutation points in LHON-iPSCs. Created in BioRender.com. **(B)** Differentiation protocol for retinal organoids (ROs). ESCM: embryonic stem cell medium, EBM: embryoid body medium, NIM: neural induction medium, RDM: retinal differentiation medium, 3D-RDM: three-dimensional retinal differentiation medium. **(C)** Staining for retinal ganglion cell (RGC) marker (magenta: Brn3), retinal progenitor cell (RPC) marker (blue: Chx10), and neural marker (green: TUJ1). Scale bars: 100 μm. **(D)** Quantification of the size of the ROs at Day35. Mean ± SEM (Cont: *n* = 46, LHON: *n* = 41).

To establish a human retinal model that mimics LHON pathology, we examined whether iPSCs derived from a control individual (non-LHON-iPSCs, 201B6 line) and a patient with LHON carrying the mtDNA mutation (11778G > A) in the ND4 gene (LHON-iPSCs) could be transformed into ROs using a modified protocol (Figures 1A,B) (Capowski et al., 2019). As in previous studies, neural retinal structures were observed on Day 14, and retinal structures were observed 27 days after differentiation. Immunostaining for Brn3 (RGC marker), Chx10 (retinal progenitor cell (RPC) marker), and TUJ1 (neural axon marker) on Day 35 revealed a layered retinal structure and the presence of RGCs, RPCs, and nerve axons (Figure 1C). No differences in RO size were observed (Figure 1D).

### 3.2 The number of RGCs and neurons is reduced in LHON-RO

**Figure 2:**
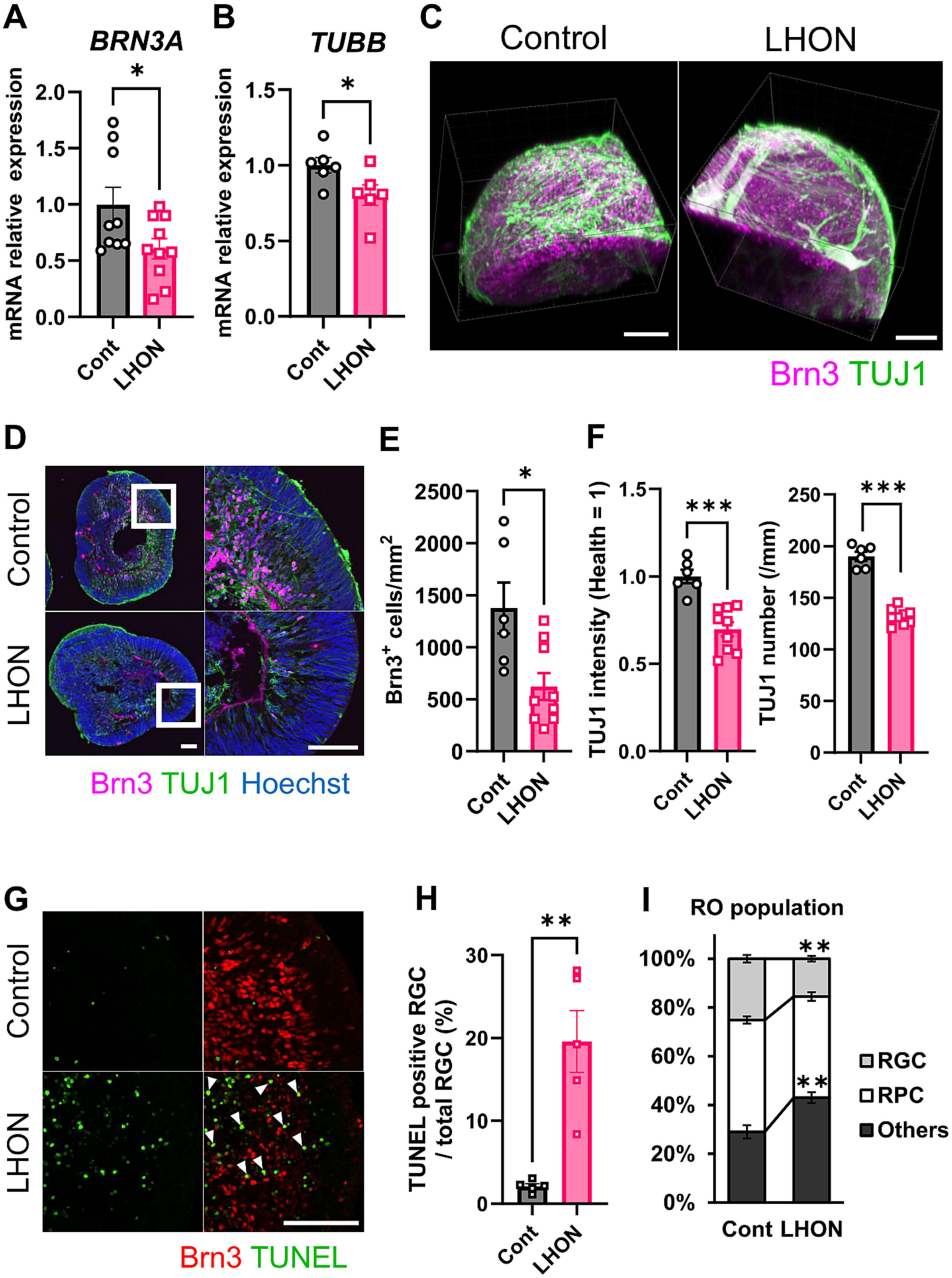
The number of RGCs and neurons was reduced in LHON-RO. **(A,B)** The expression levels of mRNA for *BRN3A* and *TUBB* were measured by qPCR of ROs at Day 35. Mean ± SEM (Control: *n* = 6–8, LHON: *n* = 8–11). Student’s *t*-test (two-tailed), *p < 0.05 (**A**: *p* = 0.045, **B**: *p* = 0.042). **(C,D)** Staining for RGC marker (magenta: Brn3) and neural marker (green: TUJ1) in Day 35 Control-RO and LHON-RO. **(C)** Whole, **(D)** slice. Scale bars: 100 μm. **(E)** The number of RGCs in the entire RO was counted. **(F)** The number of neurons was counted, and the intensity of TUJ1 expression was measured. Mean ± SEM (Control: *n* = 6, LHON: *n* = 9). Student’s *t*-test (two-tailed), *p < 0.05 (**E**: *p* = 0.011), ***p < 0.001. **(G)** Staining for RGC marker (Red: Brn3) and apoptosis marker (green: TUNEL) in Day 35 Control-RO and LHON-RO. Arrow: Brn3^+^, TUNEL^+^ cells. Scale bars: 100 μm. **(H)** The percentage of TUNEL-positive RGCs relative to the total number of RGCs was measured. Mean ± SEM (*n* = 6). Student’s *t*-test (two-tailed), **p < 0.01 (*p* = 0.0016). **(I)** Quantitative data of cell population. Results are presented as the mean ± SEM (Control: *n* = 6, LHON: *n* = 4).

In LHON patients, thinning of the RNFL has been observed, along with a decrease in RGCs and the optic nerve (Saadati et al., 1998; La Morgia et al., 2010; Grzybowski et al., 2015). To confirm these changes in LHON-RO, mRNA expression levels were compared between Control-RO and LHON-RO by qPCR on Day 35. Compared to Control-RO, LHON-RO showed a decrease in *BRN3A* (RGC marker) and *TUBB* (neural axon marker) (Figures 2A,B; Supplementary Figures 1A,B). The expression level of other RGC markers such as *POU4F2*, *RBPMS*, *THY1* and *SNCG* was decreased in LHON-RO on Day 35 (Supplementary Figure 2). Immunostaining on Day 35 and 45 showed a reduction in the number of RGCs (Figures 2C–E; Supplementary Figures 1C,D, 3A,B), decreased expression of neuronal markers, and a reduced density of nerve axons in LHON-RO compared to Control-RO (Figures 2D,F). The rate of TUNEL positive RGCs was increased in LHON-RO (Figures 2G,H). Percentage of the Chx10-positive was not changed between control-RO and LHON-RO (Figure 2I). However, LHON#2 had a low success rate of 25% in organoid formation (Supplementary Figure 1E), thus, Control and LHON#1, were used in subsequent studies.

### 3.3 Expression of mtDNA-related genes in LHON-RO and LHON-FB

**Figure 3:**
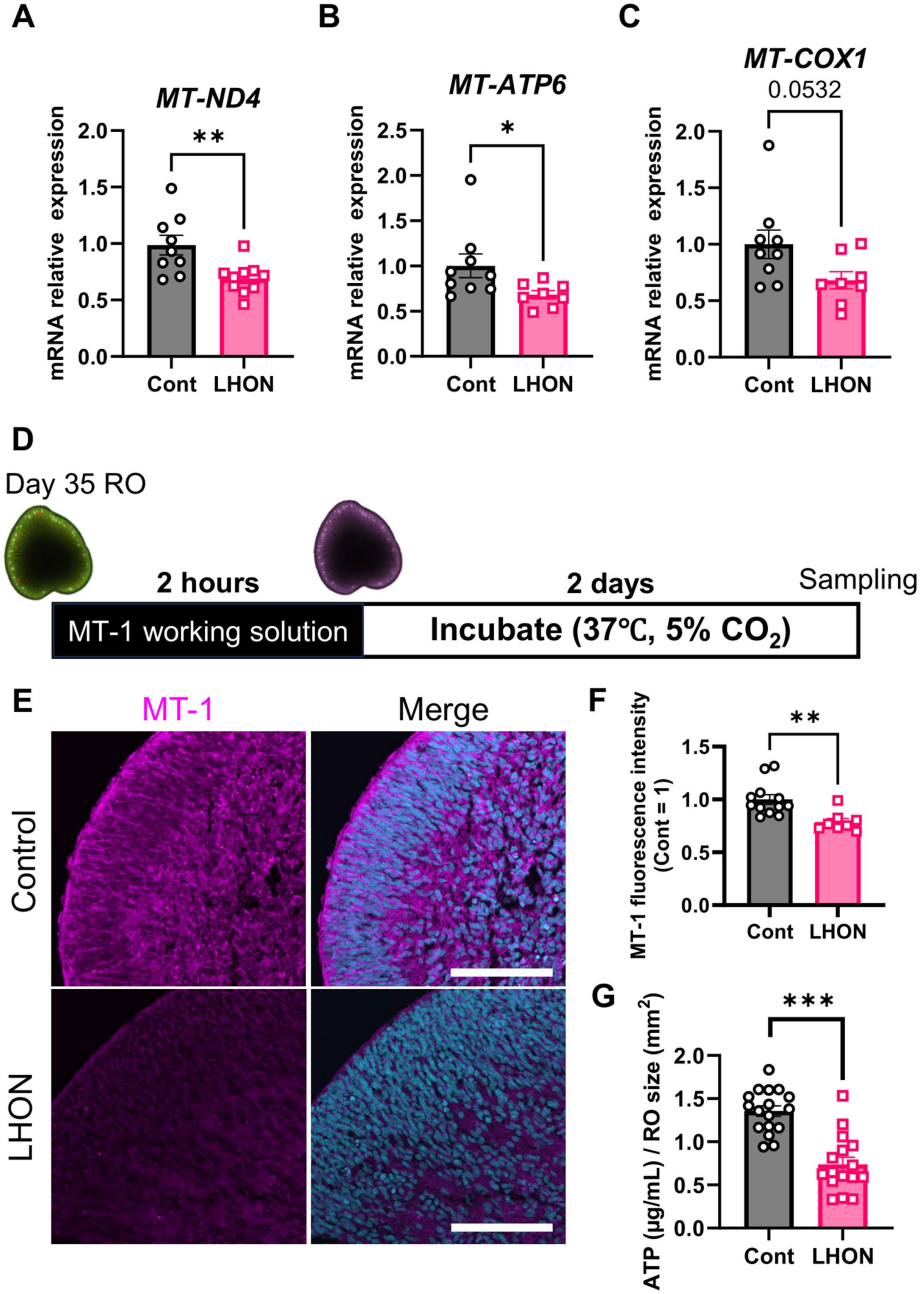
mtDNA-related genes and mitochondrial activity in LHON-RO. **(A–C)** The expression levels of mRNA for *MT-ND4*, *MT-ATP6*, and *MT-COX1* were measured by qPCR of ROs at Day 35. Mean ± SEM (Control: *n* = 12, LHON: *n* = 8). Student’s *t*-test (two-tailed), *p < 0.05 (**B**: *p* = 0.046) **p < 0.01 (**A**: *p* = 0.005). **(D)** Protocol for MT1 assay. **(E)** Representative images of Day-37 ROs stained with MT1 (magenta). **(F)** The intensity of MT1 expression was measured. Scale bar = 50 μm. Mean ± SEM (Control: *n* = 12, LHON: *n* = 8). Student’s *t*-test (two-tailed), **p < 0.01 (*p* = 0.003). **(G)** Quantification of ATP production in Day-35 ROs. Mean ± SEM (Control: *n* = 17, LHON: *n* = 16). Student’s *t*-test (two-tailed), ***p < 0.001.

Because LHON is a disease caused by mutations in mtDNA, we investigated the expression level of mtDNA. In this study, we used LHON patient iPSCs (m.11778G > A), which have a mutation in *MT-ND4*. The expression levels of mtDNA were compared by qPCR on Day 35 (Figures 3A–C). Because ND4 is a component of the mitochondrial respiratory chain complex I subunit (Zhang et al., 2019), the expression level of *MT-ND4* was decreased in LHON-RO (Figure 3A). We also examined the expression levels of the downstream factors of complex III and complex V subunits (Zhang et al., 2019). We found that both *MT-COX1*, a component of complex III, and *MT-ATP6*, a component of complex V, decreased in LHON-RO (Figures 3B,C). We also analyzed fibroblasts (FBs) from LHON patients (LHON-FB) and control individuals (Control-FB) based on gene sets associated with mitochondrial complexes. The results showed differences in gene expression between LHON-FB and Control-FB (Supplementary Figure 4A). GSEA was then performed and revealed that gene sets related to mitochondrial complexes were enriched in FBs from non-LHON patients (Supplementary Figure 4B). Abnormal mtDNA was detected in LHON-RO cells, resembling the mtDNA abnormalities observed in cells derived from LHON patients.

### 3.4 Mitochondrial activity and ATP production are reduced in LHON-RO

To confirm whether mtDNA mutations reduce mitochondrial function, the mitochondrial function of LHON-RO was examined. Mitochondrial membrane potential, an indicator of mitochondrial activity, was evaluated using the MT1 assay on Day 35. The results showed that the fluorescence intensity of the MT1 assay was decreased in LHON-RO (Figures 3D,E), indicating that mitochondrial activity was reduced. ATP production, a major function of mitochondria, was evaluated, and it was confirmed that ATP production was reduced in LHON-RO compared to Control-RO (Figure 3F).

### 3.5 Idebenone rescued the decrease in the RGC number in LHON-RO

**Figure 4:**
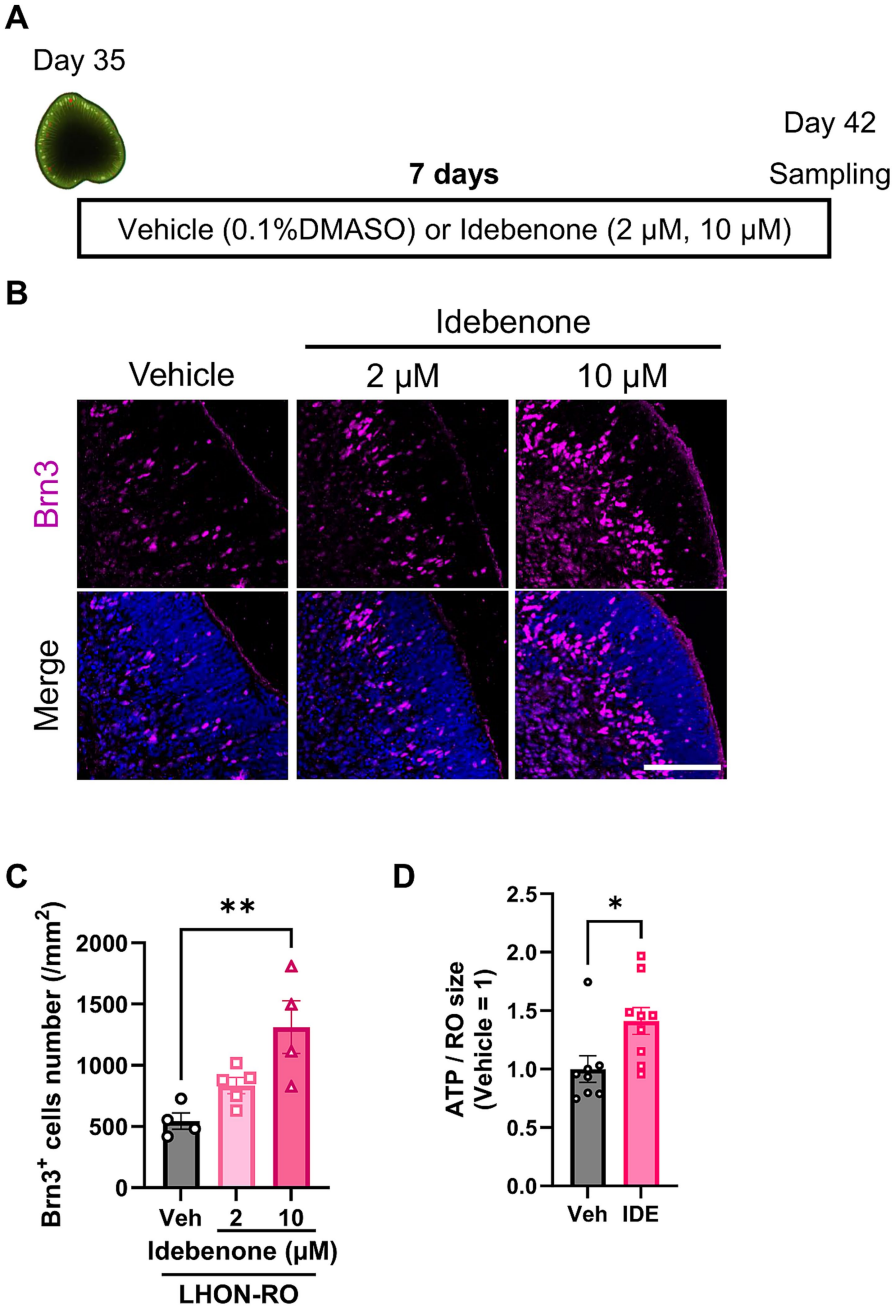
Idebenone increased the RGC number in LHON-RO. **(A)** Protocol for idebenone treatment. **(B)** Representative images of ROs treated with idebenone (2 or 10 μM) for 7 days, immunostained with BRN3 (magenta). **(C)** The number of RGCs in the entire RO was counted. Scale bar = 100 μm. Mean ± SEM (*n* = 4, 5). Dunnett’s test, **p < 0.01 (*p* = 0.004). **(D)** Quantification of ATP production in Day 42 ROs treated with idebenone (10 μM) for 7 days. Mean ± SEM (*n* = 8, 9). Student’s *t*-test (two-tailed), **p < 0.01 (*p* = 0.008).

To examine whether idebenone has a therapeutic effect in the LHON-RO model, as well as in LHON patients, LHON-RO was treated with idebenone (2 or 10 μM) for 7 days (Figure 4A). Immunostaining of slices showed an increase in the number of RGCs after treatment with idebenone (10 μM) (Figures 4B,C). Treatment with idebenone (10 μM) also increased ATP production in LHON-RO (Figure 4D). Taken together, idebenone rescued the decrease in the number of RGCs in LHON-RO by increasing ATP production.

### 3.6 Mitophagy was dysregulated in LHON-RO

**Figure 5:**
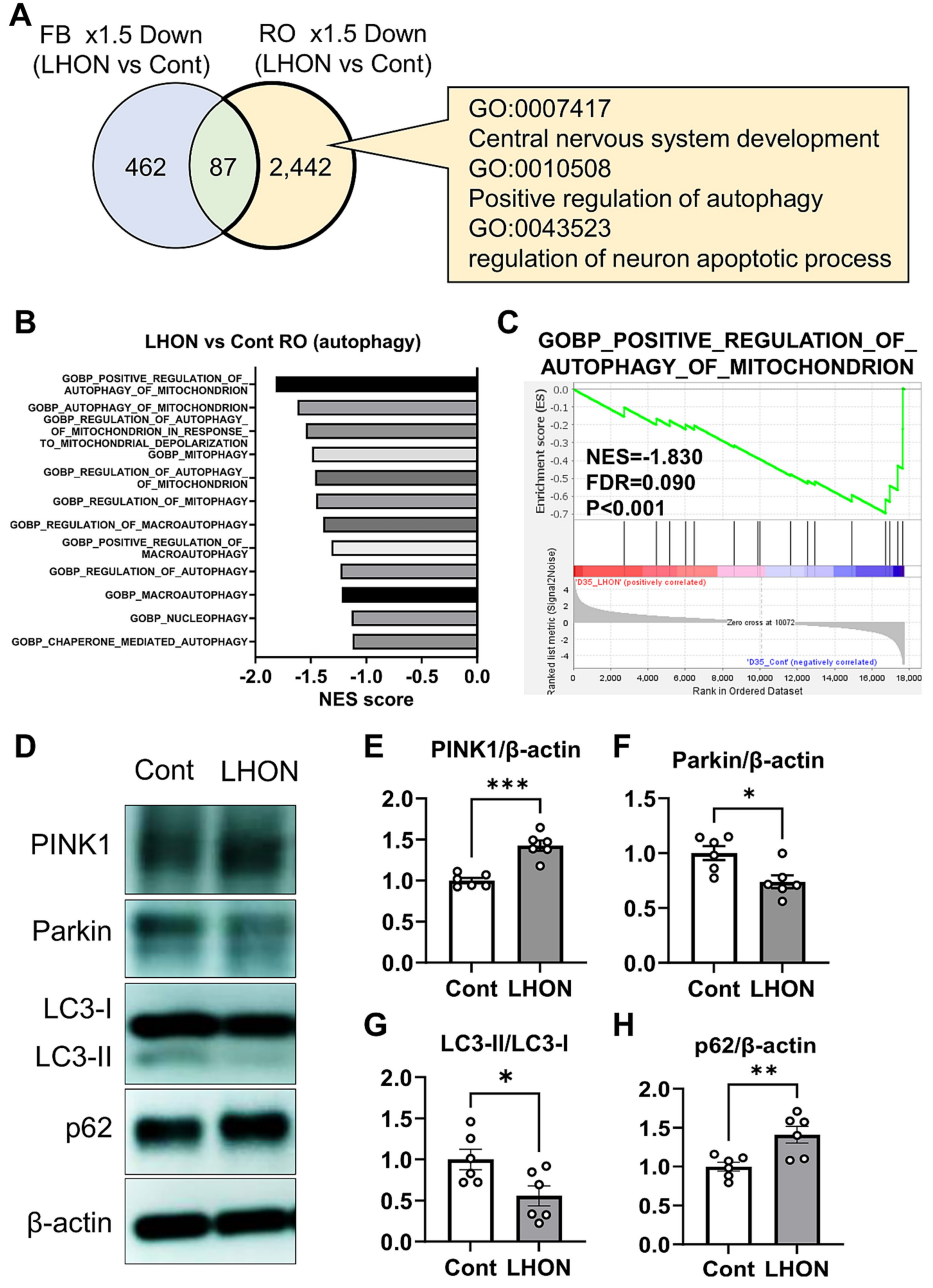
Mitophagy was reduced in the LHON-RO model. **(A)** Extraction of enriched genes in LHON-RO not LHON-FB, as shown in the Venn diagram. Among the differentially expressed genes, overexpressed gene ontology (GO) terms were identified. **(B,C)** Gene set enrichment analysis of autophagy related genesets in LHON-RO. **(D)** Control RO and LHON RO was analyzed by Western blotting. The representative immunoblots show the expression of PINK1, Parkin, LC3-I, LC3-II, p62 and *β*-actin in the cell lysate of RO. **(E–H)** Quantitative analysis of PINK1 **(E)**, Parkin **(F)**, LC3-II/LC3-I **(G)** and p62 **(H)** expression. Mean ± SEM (*n* = 6). Student’s *t*-test (two-tailed), *p < 0.05, **p < 0.01 ***p < 0.001 (**E**: *p* = 0.0001, **F**: *p* = 0.0136, **G**: *p* = 0.0290, **H**: *p* = 0.0077).

To clarify the pathogenesis of LHON, RNA-Seq was performed using LHON-RO. As a result, 2,529 DEGs were identified as genes that were reduced by more than 1.5-fold compared to Control-RO (Figure 5A). Comparing the DEGs of the LHON-FB with LHON-RO, many of them were not overlapped (Figure 5A). Further GO analysis was performed using DEGs of the unmatched regions (decreased in LHON-RO and unchanged in LHON-FB) and 352 GOs were found, including GO:0007417 Central nervous system, GO:0010508 Positive regulation of autophagy, GO:0043523 Regulation of neuron apoptotic process, and GO:0043523 Neuron apoptosis. GO:0043523 Regulation of neuron apoptotic process (Figure 5A). GSEA analysis of the LHON-RO data set showed that autophagy-related gene sets, especially mitophagy-related gene sets, were enriched in Control-RO (Figures 5B,C). To demonstrate the impairment of mitophagy in LHON-RO, mitophagy markers were evaluated by Western blotting. We evaluated the mitophagy-related proteins; PINK1 labels defective mitochondria involved in initiating mitophagy, Parkin is a ubiquitin ligase and is involved in mitophagy initiation by activating PINK1, the ratio of LC3-II/LC3-I means autophagosome formation, p62 is a protein that acts as a receptor linking depolarized mitochondria to LC3 on autophagosomes and is degraded by lysosomes. Changes in these markers suggested that mitophagy may not work properly (Mizushima and Yoshimori, 2007; Pickles et al., 2018). The results showed an increase in PINK1, a decrease in Parkin, a decrease in LC3-II/LC3-I ratio, and an increase in p62, indicating the impairment of mitophagy, in LHON-RO (Figures 5D–H). Taken together, we suggested that the process of mitophagy could be involved in the pathology of LHON-RO.

## 4 Discussion

In this study, we successfully established a human retinal model using LHON patient-derived iPSCs. LHON-RO showed a reduction in the density of RGCs and their neural axons compared to non-LHON-RO. The expression level of *MT-ND4*, which indicates the mtDNA mutation 11778G > A in LHON patients, was also decreased in LHON-RO. In accordance with this result, mitochondrial membrane potential and ATP production were also decreased. Finally, we showed that idebenone rescued the decrease in the number of RGCs in LHON-RO. Our data suggest that the LHON-RO model is a valuable system for evaluating drug efficacy.

The differentiation protocol for LHON-RO was established in this study, referring to protocols from previous studies (Capowski et al., 2019). LHON-RO showed a layered structure of the retina, which is characteristic of ROs (Capowski et al., 2019; Li et al., 2021). In this study, we found that the number of RGCs and the density of neural axons were decreased in LHON-RO, and it was due to increased cell death (Figures 2G,H). Clinically, RNFL thinning (a decrease in RGCs and optic nerve axons) has been observed in LHON patients (Saadati et al., 1998; La Morgia et al., 2010; Grzybowski et al., 2015). Therefore, the reduction in the number of RGCs and the density of neural axons, key clinical findings in LHON patients, was recapitulated in LHON-RO. While, LHON#2-iPS is not well to differentiate into ROs (Supplementary Figure 1E). This could be due to low colony-forming ability of iPS cells (data not shown) which is caused by more severe condition of LHON#2 patient.

ROs were generated using iPSCs derived from an LHON patient with the mtDNA (1778G > A) mutation in the *ND4* gene, the most frequent mutation in LHON patients (La Morgia et al., 2010). Our data showed that the expression levels of *MT-ND4* (mitochondrial complex I), *MT-COX1* (complex III), and *MT-ATP6* (complex V) were decreased in LHON-RO (Figures 3A–C). In Supplementary Figure 4, genes related to mitochondrial complexes, especially complexes I and V, are decreased in LHON-FB. The decreased expression of *MT-ND4* was also observed in LHON-iRGCs, similar to a previous report (Wu et al., 2018). Thus, the LHON-RO model can recapitulate mtDNA abnormalities in LHON patients. The decreased expression of complex I may have also affected the expression levels of complexes III and V, which are downstream of the electron transfer system. However, compared to complex I, the difference in the expression of complexes III and V may have been small because it was an indirect effect. It is suggested that the decreased expression of mtDNA has some effect on the mitochondrial electron transfer system and other functions (Distelmaier et al., 2009; Fassone and Rahman, 2012). In fact, our results indicated a decrease in mitochondrial function in LHON-RO (Figures 3E–G). Previous studies have shown that mitochondrial respiratory capacity and ATP production are decreased in LHON-iRGCs and FBs (Wu et al., 2018; Zhou et al., 2022; Nie et al., 2023). These results suggest that the decrease in RGCs and nerve axons observed in this model may be due to reduced mitochondrial function. Two factors, genetic and environmental, are known to contribute to the pathogenesis of LHON. In our model, LHON phenotypes (decreased RGC number and axon density) were observed during differentiation into ROs. Thus, the culture environment or cell–cell interactions in the RO may be involved in the pathogenesis of LHON, and future studies could accelerate the understanding of LHON pathogenesis.

Currently, idebenone is the only drug approved for the treatment of LHON (Mashima et al., 1992; Klopstock et al., 2011). Idebenone is a COQ10 analog that improves mitochondrial function by affecting the mitochondrial electron transfer system (Haefeli et al., 2011). In a previous study, idebenone treatment suppressed cell death in LHON-FBs (Danese et al., 2022). In transmitochondrial cybrids with LHON-specific mtDNA mutations, idebenone treatment also suppressed cell death and enhanced oxygen consumption rate, an indicator of mitochondrial function (Aleo et al., 2024). However, these reports did not show the protective effect of idebenone against the death of RGCs in the retina. We demonstrated that idebenone prevented the reduction of RGCs in LHON-RO (Figures 4B,C). Our retinal model is potentially applicable for recapitulating human pathological mechanisms, such as RGC-other neurons or RGC-glia interactions in LHON patients. In summary, the establishment of a retinal model that more accurately mimics the pathophysiology than the conventional FB model has made it possible to evaluate the efficacy of idebenone.

Our data showed that mitophagy was dysregulated in LHON-RO (Figure 5B). Several studies have reported increased mitochondrial mass or mtDNA in cells with LHON mutations (Korsten et al., 2010; Giordano et al., 2011, 2014; Bianco et al., 2017), affecting mitochondrial homeostasis which reflects the balance between mitochondrial biogenesis and mitophagy (Herzig and Shaw, 2018). In other words, changes in mitochondrial mass or mtDNA copy number by LHON mutations can cause abnormalities in mitophagy, which is a target based on the pathogenesis mechanism of LHON. Although multiple studies have examined mitophagy in LHON, the effect remains controversial; some studies demonstrated the impairment of mitophagy (Zhang et al., 2018, 2021; Liang et al., 2022), while other studies suggested the enhancement of it (Dombi et al., 2016; Danese et al., 2022). However, in both cases, the LHON patient-derived FBs or cybrids (LHON-mitochondria transplanted cells) were used. Since LHON is a retina-specific pathology in most cases (Levin, 2007), our RO model could properly reflect the pathology of LHON. In addition, since the accumulation of defective mitochondria is known to induce cell death through the generation of mtROS, etc., (Danielson et al., 2002; Zanna et al., 2005), mitophagy has potential as a therapeutic target on LHON.

In conclusion, the LHON-RO model has proven to be a useful system for elucidating the pathogenesis of LHON and evaluating drug efficacy. In particular, LHON-RO showed a decrease in RGCs and nerve axons, mitochondrial dysfunction, and responsiveness to idebenone, a therapeutic agent. Furthermore, a comprehensive analysis of the LHON-RO model revealed a reduction in mitophagy, potentially leading to the development of novel LHON therapeutics targeting mitophagy.

## Data Availability

The datasets generated in this study are available on the National Center of Biotechnology Information (NCBI) Gene Expression Omnibus (GEO) database at (accession number: GSE305550) https://www.ncbi.nlm.nih.gov/geo/query/acc.cgi?acc=GSE305550. The data that support the findings of this study are available from the corresponding author upon reasonable request. RNA-seq dataset of FBs from LHON patients and non-LHON patients was available in NCBI Gene Expression Omnibus (accession number: GSE144914).

## Footnotes

1
http://www.broadinstitute.org/gsea/downloads.jsp
