# Supplementary figures and images for: Establishment of human Leber’s hereditary optic neuropathy model using iPSC-derived retinal organoids
Source: Front Cell Neurosci. 2025 Sep 12;19:1635775. doi: 10.3389/fncel.2025.1635775 (PMC12464001; doi:10.3389/fncel.2025.1635775)

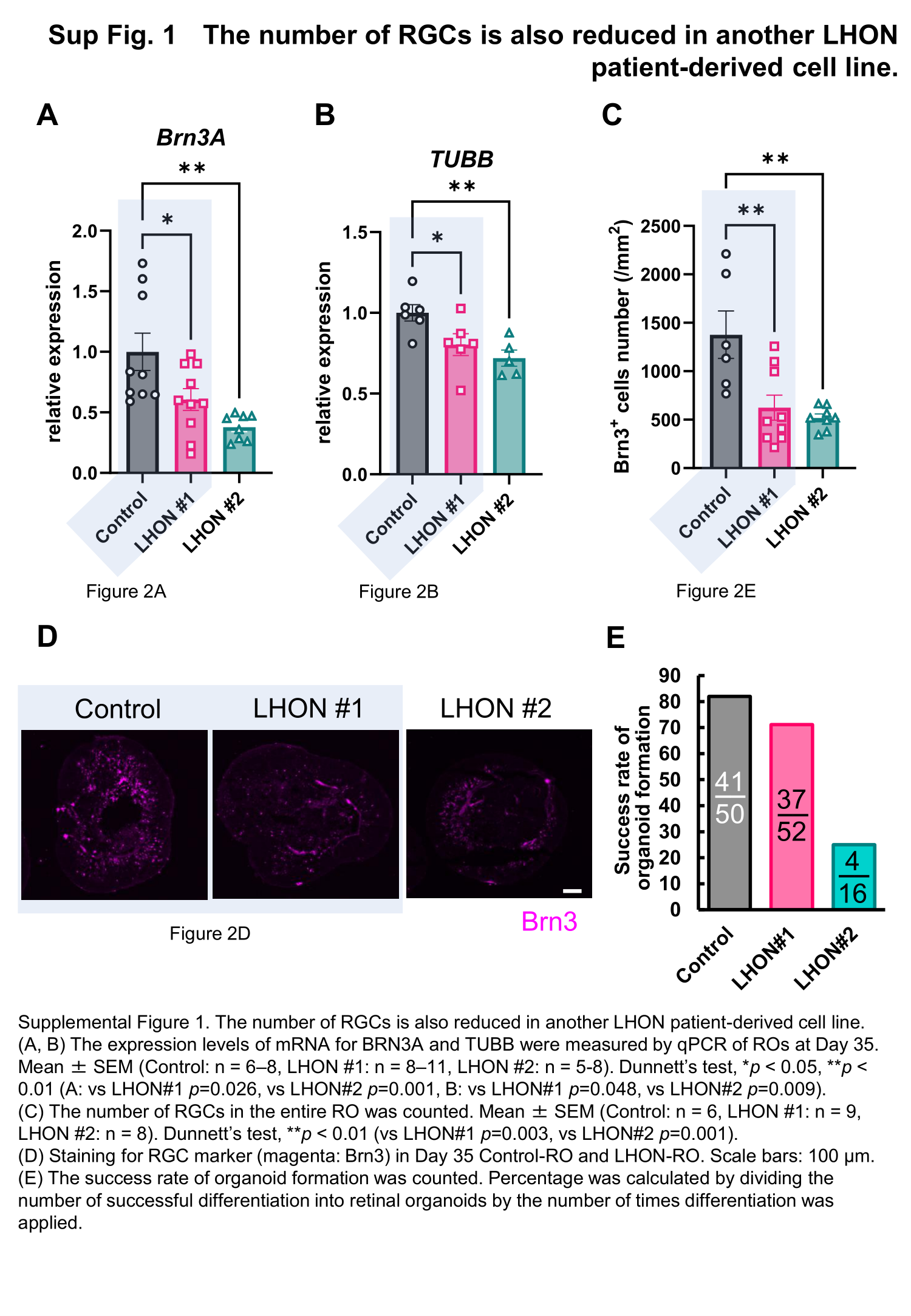

Supplement: Supplementary file 1 [file Image_1.TIFF]

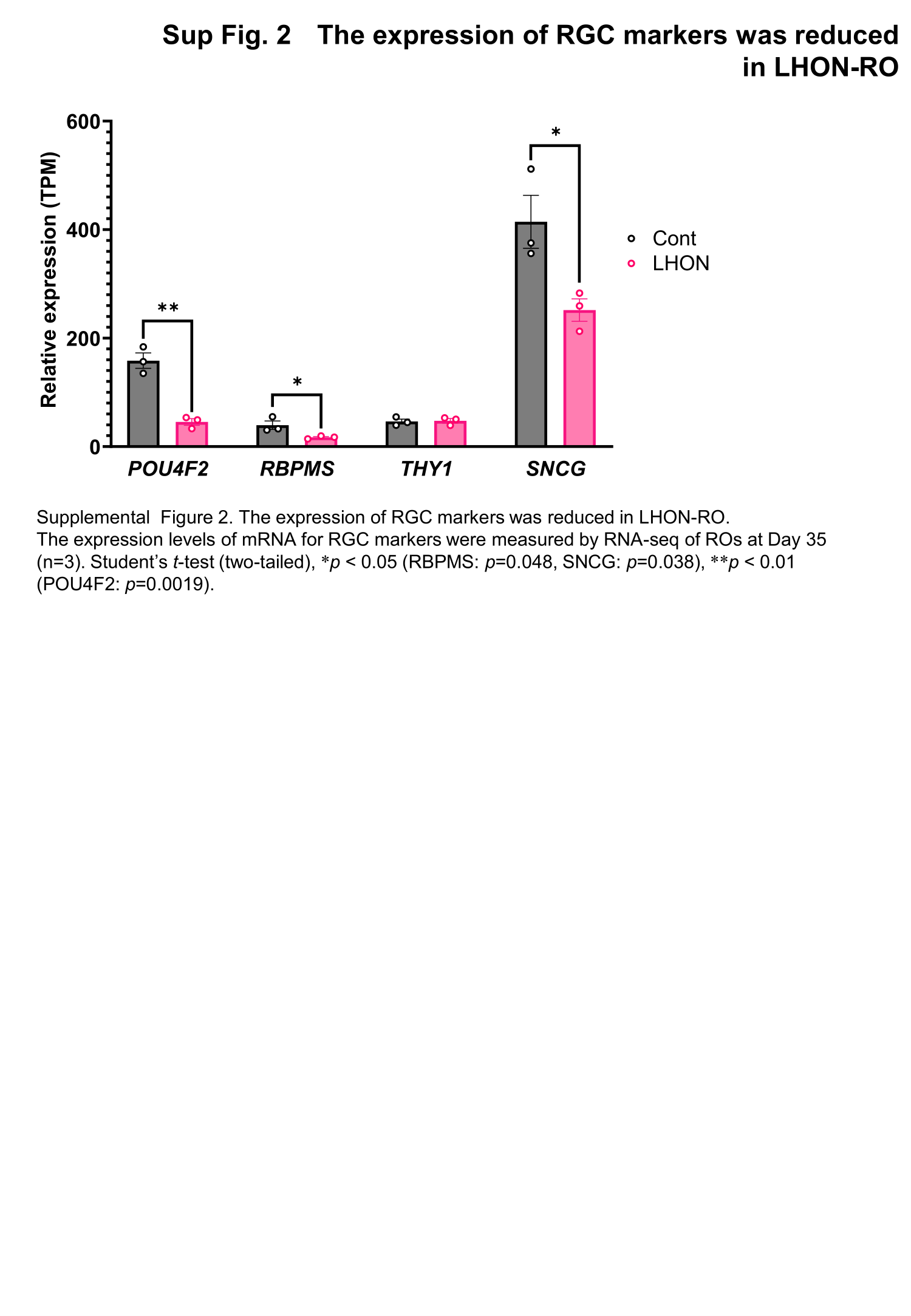

Supplement: Supplementary file 2 [file Image_2.TIFF]

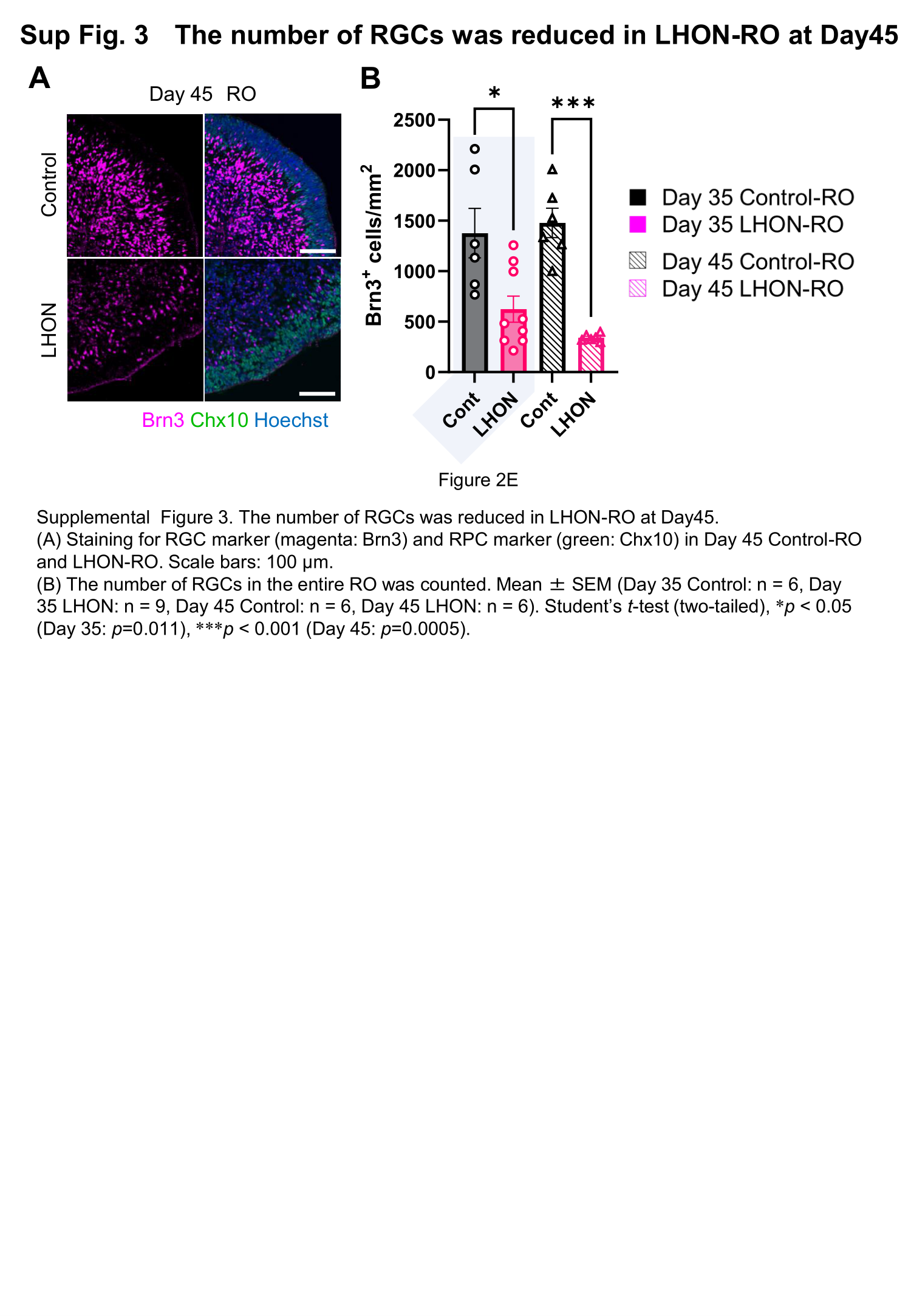

Supplement: Supplementary file 3 [file Image_3.TIFF]

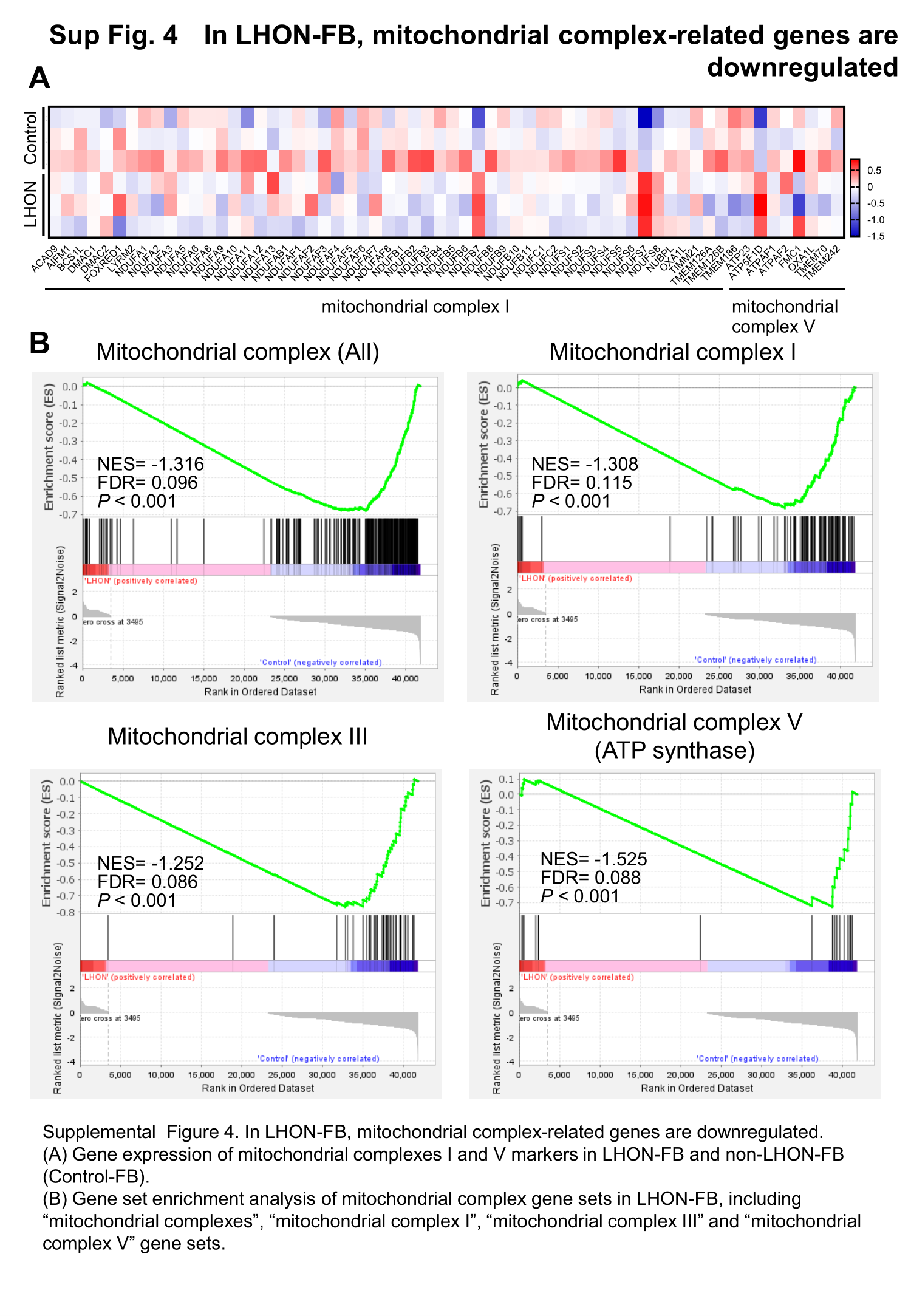

Supplement: Supplementary file 4 [file Image_4.TIFF]
